# Family carers’ experiences of serious illness conversations in haematological malignancies – a qualitative interview study

**DOI:** 10.1186/s12904-026-02214-w

**Published:** 2026-07-04

**Authors:** Isabel Hansson, Malin Bengtsson, Stina Nyblom, Joakim Öhlén, Lena von Bahr

**Affiliations:** 1 Section for haematology and coagulation, Sahlgrenska University Hospital, Gothenburg, Region Västra Götaland Sweden; 2 Institute of Health and Care Sciences, Sahlgrenska Academy, University of Gothenburg, Gothenburg, Sweden; 3 Palliative Centre, Sahlgrenska University Hospital, Gothenburg, Region Västra Götaland Sweden; 4 Centre for Person-Centred Care (GPCC), University of Gothenburg, Gothenburg, Sweden; 5 Institute of Medicine, Sahlgrenska Academy, University of Gothenburg, Gothenburg, Sweden

**Keywords:** Family carer, Haematological malignancies, Palliative care, Serious illness conversations, Caregivers

## Abstract

**Background:**

Family carers of cancer patients commonly experience anxiety, as well as grief. To better manage their situation, they seek to understand the disease process and become involved in decision-making. Early integration of palliative care and serious illness conversations have been shown to improve quality of life, reduce symptom burden, and support both patients and their families. In haematological malignancies, patients often experience high disease burden, poor quality of life, and aggressive treatment near the end of life, with end-of-life discussions frequently occurring too late. This may impact on the experience of their family carers.

The aim of this study was to explore family carers’ experiences of communication about serious illness and palliative care in the context of haematological malignancies.

**Method:**

This study has a qualitative descriptive design. Family members of patients who had died with haematological malignancy, treated at a University Hospital in Sweden, were invited to participate. A total of 9 family carers were interviewed between May 2023 and October 2024.

**Results:**

Three main themes were identified in the experiences of the interviewed family carers: (1) Feeling excluded but still responsible, (2) Having time to prepare, and (3) Talking about difficult things.

**Conclusions:**

The study shows that family carers of patients with haematological malignancies shoulder great responsibilities while simultaneously often feeling excluded from important conversations. The study highlights the need to include family carers and introduce conversations about palliative care early on, in order to provide support and facilitate communication between patient and family.

**Supplementary Information:**

The online version contains supplementary material available at 10.1186/s12904-026-02214-w.

## Background

Treatment of haematologic malignancies has undergone tremendous improvement in recent years, providing dramatic responses to diseases previously deemed incurable [1, 2]. However, patients remain a heterogeneous group, with high morbidity and a wide variation in disease course and prognosis [3].

Commonly, patients with haematologic malignancies have a high disease burden and poor quality of life. They often receive aggressive treatment, even at the end of life, and are more likely to die in hospital [4]. Previous studies have shown that end-of-life discussions are often initiated too late for these patients [3, 5] and palliative care is often discussed only when death is imminent. Communicating prognosis and goals of care in the setting of unpredictable treatment response is challenging but all the more important to help patients and their family carers navigate a tumultuous situation. Studies on earlier integration of palliative care have shown increased quality of life and a reduced symptom burden in patients [6], as well as improved well-being for family carers [7]. The purpose of ‘serious illness conversations’ is to allow patients and their families to express their own thoughts, wishes and priorities, while at the same time providing them with the information they want about their current condition and expected future course [8, 9]. Identifying when to initiate these conversations is a complex task, and fear of taking away hope from patients can delay the process, especially when health care professionals outside of specialised palliative care are involved [10].

Family carers of patients with haematological malignancies report high levels of anxiety, grief and poor quality of life [11, 12]. They tend to search widely for information regarding prognosis and treatment as a means of regaining control and helping them provide care [13]. Including family carers in shared decision making is recommended in cancer practice and can reduce the burden experienced by the family [14]. Consequently, serious illness conversations may be one tool which can help include family members in communication [15]. Before a transition to palliative care has been made, uncertainty can lead to significant suffering for both patients and family carers [16]. It is therefore important to navigate this carefully in communication. Once a transition to palliative care has been made, more open communication concerning end-of-life care is possible [17]. However, in haematological malignancies, this transition tends to happen late in the progression of the disease, if at all [3, 5]. Consequently, if our objective is better inclusion of family carers, it is important to understand how they perceive and receive communication throughout the disease trajectory.

Little is known about the practical experiences of illness communication from the perspective of family carers of patients with haematological malignancies. Previous studies on conversations in serious illness have mainly focused on conversations between patients and physicians. A few studies exist on the experiences of family carers of patients treated for haematological malignancies [13, 18] but no studies specifically concerning their experiences of communication with healthcare providers.

The aim of this study, therefore, is to explore the experiences of family carers of patients who have died with haematological malignancies, especially in regard to their communication with healthcare professionals about serious illness, palliative care and end-of-life discussions, from diagnosis to the end.

## Methods

### Design and participants

This study was designed as a qualitative explorative study, following EAPC recommendations on palliative care research with family carers [19]. The report follows the COREQ 32-item checklist for reporting qualitative data [20].

Participants were family carers of deceased patients with haematological malignancies. The sampling principle was consecutive sampling during the study period. Patients whose death was deemed unexpected or who had requested confidentiality in relation to family carers were excluded. Participants were recruited via an information letter, which was sent to the address of the deceased patients who had received treatment for haematological malignancies at the Sahlgrenska University Hospital haematology clinic in Gothenburg, Sweden. The letter was sent after a minimum of 6 weeks had passed from the death of the patient and contained a pre-paid return acceptance letter for interested participants. No reminder letters were sent. Consequently, the sample size was ethically and pragmatically limited as we did not want to bother bereaved family members with reminders. Due to this, data saturation was deemed unlikely and was not an aim of the study.

Family carers who agreed to participate were contacted by telephone to arrange a time and place for the interview. Participants were free to choose the location of the interview.

### Research team

Interviews were conducted by MB (female registered nurse with palliative care experience and PhD student) and IH (female medical student performing a master thesis) under guidance from JÖ (male registered nurse, professor in palliative care and experienced qualitative researcher). Data analysis was performed by IH and LvB (female Md PhD specialised in haematology and palliative care), with the codes and themes discussed in the entire research team.

### Data generation

Data were generated through semi-structured interviews using an interview guide. The introduction to the interview was phrased as follows: *“We will talk about conversations you had about your family member’s illness*,* about their care and about how you and your family member thought about the future. Would you like to tell us about conversations you had with health professionals about your family member’s illness?”*

Further questions were asked to explore the topics of serious illness conversations and palliative care, if they were not spontaneously raised by the respondent. The term ”serious illness conversation” was not expressively used in the interviews, as this has not been a generally used term in swedish health care during the studied period, but rather the concept was explored by asking questions regarding conversations about future, forecasting and the wishes of the patient. For palliative care, the respondents were asked about what this term meant to them.

The intervew guide was initially developed for patient interviews. The questions were constructed by the research group in collaboration with a patient representative, and pilot tested in 3 patient interviews, after which some minor changes to the phrasing were made. Adaptation to the family carer perspective was made by the research team, without changing any topic of the questions. No further pilot testing was done. Details of the guide can be found in Supplementary 1. Demographic information was collected at the beginning of the interview.

The first four interviews were conducted by researcher MB and the following five by researcher IH. All interviews were conducted face to face, primarily in the participants’ homes, apart from in two instances where the participants preferred to meet at the hospital. All interviews were audio-recorded and then transcribed verbatim and pseudonymised.

### Data analysis

The data was analysed according to Malterud’s method of systematic text condensation [21] – a method based on Giorgi’s psychological phenomenological analysis [22]. No software was used in the analysis.

In step 1, researchers IH and LvB separately read through all the interviews to get an overall picture and identified preliminary themes. These were then discussed in the research team, and four preliminary themes were agreed on for further analysis. The subsequent analysis was initially done by researcher IH, with frequent discussions with LvB.

In step 2, the first three interviews were reviewed in depth to identify meaning units, which were then extracted and coded. Codes were then arranged in code groups based on the inductively identified preliminary themes. In step 3, the meaning units of each subgroup were abstracted into a condensate written in first person format.

Steps 2 and 3 were then repeated for interviews 4–6 and 7–9, respectively. After each round of analysis, the resulting code groups and condensates were compared to the previous ones and merged, and when new codes or groups emerged, the previous material was reviewed and re-coded. For each code group, a quote was selected as an example to illustrate the meaning.

Finally, an analytical text was written for each code group, synthesizing the meanings into themes based on the condensed data. The results were then validated through recontextualization, where the new meaning descriptions and themes were compared to the entire original data. The resulting themes were not shown to informants for feedback before publishing.

### Ethical approval

The study follows the principles of the Declaration of Helsinki and was approved by the Swedish Ethical Review Board, Dnr 2023-00984-02. All participants signed informed consent.

## Results

In total, the request was sent out to 36 family carers, 10 of whom chose to participate. One of these could not be reached by telephone to book an interview, despite repeated attempts to contact them. The study consequently included 9 interviews with 4 men and 5 women aged 34–87 years (median age 71 years). The characteristics of the participating family carers and their diseased family member are detailed in Table 1. All patients were continuously followed at the haematology clinic at Sahlgrenska University Hospital and had episodes of emergency hospital care in the last months of life.

**Table 1:** Demographic information on family carers and patients

	Family carers	Patients
Sex	4 men	4 men
	5 women	5 women
Age (median)	34–87 years (71)	56–89 years (78)
Relationship to patient	7 partners	
	2 children	
Educational background	2 secondary school	2 secondary school
	1 high school	4 high school
	6 college/university	3 college/university
Country of birth	9 Sweden	9 Sweden
Diagnosis		5 Lymphoma
		2 Myeloma
		1 AML
		1 Blood cancer unspecified
Place of death		4 Haematology ward
		1 General medicine ward
		1 Hospice
		1 Own home (with palliative team support)
		2 Unclear

Three main themes were identified in the experiences of the interviewed family carers: (1) Feeling excluded but still responsible, (2) Having time to prepare, (3) Talking about difficult things.

### Feeling excluded but still responsible

Several family carers felt that the healthcare professionals mainly addressed the patient in conversations. At the same time, the family carers experienced difficulties in communicating directly with the patient, suggesting that the patient kept information from them in an effort to protect them. Nevertheless, the family carers felt that a lot of responsibility had been placed on them in terms of practical care, keeping track of appointments and medications, and communicating with the extended family.

This combination of being kept in the dark while having great responsibility left family carers feeling lonely and abandoned. Their vulnerability was described mainly as a sense of powerlessness and of being in someone else’s hands, as described by one participant:

*“All the while you wonder what’s happening and feel very vulnerable. It’s like you’re sitting in an aeroplane – you’re in the hands of the pilot up front and can’t do anything yourself. And then*,* of course*,* there’s this feeling of being abandoned*,* quite simply. There’s an emotional storm inside of you.”* (Interview 5)

Some family carers would have liked a more proactive approach from healthcare staff – that they would have taken the initiative to ask them how they were doing and invited them to speak. Other family carers wanted more psychosocial support, stating that healthcare staff should suggest contact with a counsellor early on, as realising the need and addressing it yourself can be difficult.

The field of haematology was perceived as very complex. Some family carers prepared for the doctor’s appointments at home by discussing what to bring up and what questions to ask. In cases where the patient had reduced energy, the family carers felt responsible for speaking for them and leading the discussion. Several participants also felt that they were responsible for updating other family members on the current status.

As all patients were immunocompromised, most participants described susceptibility to infection as a major concern. This brought responsibilities in the form of restrictions on everyday life, such as reduced socialising and special handling of food. One participant said this led to a great deal of conflict at home, as family members had different views on how much to restrict themselves. In cases where the patient had an infection, several family carers described concerns about whether they had been the source of infection. They ruminated about whether they should have done something differently. One participant said:

*“He had been given leave – how were we to relate to that? I didn’t know*,* of course – when your immune system is as weak as his was. What are we going to…like*,* I ordered some prawns*,* we were going to eat*,* were we allowed to do that? I had a plant that smelled of fungus. I thought*,* that – that can’t be good for him. If he comes home*,* what do I do? Or what are we going to do to make sure everything is as good as it can be? Had something happened here? Because it did – when he was home on leave. Then he had to go back to the hospital at 11 am. At 2 pm he got really bad again and then I thought*,* did something here at home do this? I didn’t know.”* (Interview 7)

Difficulties in communicating with the patient were described by several family carers, who felt that patients protected them by minimising or hiding deterioration and not communicating how bad the situation actually was. Later, once they realised the truth of the situation, the family carers were shocked. One participant described this as follows:

*“And then at some point it came as such a shock to me*,* because I wasn’t aware he was that bad because he hadn’t said so – he’d hidden it. I was so shocked – it was almost like death being confirmed.”* (Interview 3)

Protection was also described in the other direction, with the family carers protecting the patient by avoiding difficult topics or issues that might worry them. One reason for this was the view that communicating openly about the end and accepting the truth about prognosis could shorten the disease trajectory, and was therefore to be avoided.

### Having time to prepare

Many family carers described being surprised by the rapid course of the disease and unprepared for what would happen. Some said if they had better understood how fast things might change, they would have prioritised things differently. Several participants described the time between having been told treatment would be discontinued and the death of the patient as being very short. Due to the rapid course of events, some family carers felt they did not have time to say a proper goodbye. In the words of one participant:

*“It happened so quickly. I never got the opportunity to say goodbye or anything – now he was just gone. I find that situation hard to grasp. From believing he was actually going to make it to becoming critical very fast.”* (Interview 2)

Some family carers did participate in conversations to plan for the future. For example, they discussed the possibility of palliative care at home or going to a hospice. Some were given plans and routines for potential future situations, which was perceived as positive. Several family carers planned for what would happen when they were alone. They prepared by learning practical chores that had previously been handled by the patient and by learning about what needs to be done after the death of a family member. Having done so, several participants said it made them feel good afterwards, for example, simply knowing how the patient wanted their funeral made it easier for the family to plan it. One family carer was worried about finances, but after considering some practical steps, reflected it was good to have some control over this beforehand and plan for the worst to avoid a lot of anxiety. One participant described their practical preparations in this way:

*“I was there when the doctor said that it’s about 65% chance that you will make it*,* and it’s very serious. I understood where we were heading when Christmas was approaching*,* and I felt that now I will be left alone. We worked a lot together in the house and I learned to cook*,* clean and turn on the washing machine. I’m glad that we did that” Interview 1*

Family carers perceived haematological diseases as very difficult and complex. Several felt doctor’s consultations focused mainly on the present treatment and short-term follow-up. Once family carers had been informed of the initial diagnosis, they would have liked more clarity about the prognosis and information about the future to prepare themselves for what was to come. A number of participants felt that the initial news had come as a shock, and that having a follow-up conversation shortly thereafter would have been a good idea.

The family carers described having limited knowledge about palliative care. Several were of the opinion that the patient had been so stable in their illness that palliative care had not been discussed. For this reason, they would have liked earlier discussions focusing on palliative care and information about its purpose.

### Talking about difficult subjects

Participants mentioned several aspects of communication with health care staff had been helpful, while perceiving some as negative.

One aspect family carers mentioned was that they appreciated the regular contact they had with physicians to review the patient’s current status, as this helped them feel well-informed. Receiving information in writing was also perceived as reassuring. Moreover, being present at patient visits was considered valuable because family carers would get the same information as the patient and also be involved in the discussion. However, one negative aspect was that family carers felt health professionals did not proactively invite them to participate in the discussions. In this regard family carers felt they needed more support – they wanted to feel included and share their views.

Family carers with experience of a long disease trajectory said they were mostly involved at the beginning and at the end – something that proved to be negative, as described by one participant:

*“During the first year I was totally in the loop but later*,* once we got into a kind of normality – everyday life with treatments – then I wasn’t involved at all. I hadn’t a clue apart from when he said he was going for a check-up or follow-up and then that was fine. Because you realise it’s really good to have two of you there – to hear [what they say]*,* ask questions and listen. It must have been because I didn’t know what was happening*,* it came as a shock before Christmas when everything was so bad because I’d been way out of the loop with the exact situation and status.”* (Interview 3)

For some family carers, being seen by the care staff felt reassuring. Care staff demonstrated that they saw family carers by asking how they were feeling and how they were doing. Family carers described the importance of feeling listened to and treated with respect. They also appreciated when healthcare providers gave suggestions based on their previous experiences. For example, they might suggest psychosocial counselling, an overnight stay in case of deterioration, or providing information to other family carers. One participant said:

*“The doctor called me when I was at home here and said*,* we’re moving him to a private room*,* so you can be here a bit more. He was weak and fragile and they said you can be here tonight*,* if you want. So I went and afterwards it felt good that I was there.”* (Interview 8)

Several family carers described how they tried to maintain hope until the end, despite deterioration and a poor prognosis. Even if they saw that the treatment was not helping and that the disease was progressing in the wrong direction, they did not want to absorb this information but instead continued to hope that things would work out. Previous experiences of turning things around from bad situations helped to maintain hope. The fact that a lot of research was being done and that there were treatments to try also made them hopeful. Several participants said that when discussing treatment, there was only one option. Despite the great risks of the treatment, they put their hope in it because they felt it was the only chance they had. Some then felt disappointed because they thought that health professionals had raised false hopes by being overly positive about a treatment that turned out to be ineffective.

Family carers perceived *Palliative care* and *hospice* as very loaded words, with several participants equating palliative care with the end. Only one family carer said that palliative care could be described in terms of hope. In all other cases, hope was clearly linked to improvement and possible new treatments. Some participants found it upsetting when the possibility of hospice care was discussed with the patient, as this gave the patient insight that death was near. Regarding doctor’s conversations towards the end, some family carers described them as mostly difficult and did not feel they did much good. Sitting and waiting for news was very psychologically stressful. One participant expressed a negative experience in this way:

*“One really stupid thing that happened was that the doctor who came and spoke to him said: Oh dear*,* I can see you’re much*,* much worse than when I last saw you. And that was true but not something that made any difference. It just made us even lower.”* (Interview 3)

Some participants had experienced what they perceived as “clumsy” comments from the healthcare staff in conversations and decisions about palliative care and hospice care. They felt that on these occasions, the healthcare staff expressed themselves in a way that psychologically harmed them rather than supported them.

## Discussion

The findings from this study show that many family carers felt excluded from communication by both the healthcare professionals and the patient. When family carers are not given the opportunity to participate in conversations, a great responsibility falls on the patient to pass the information on. An important finding in this study was that in many cases the patient was not able to do this. Several explanations for this result are possible. The carers felt it was due to the patient wanting to protect them, for example, by hiding deterioration of current status, and this is supported in the literature [23]. Other possible explanations could be that the patient was unable or unwilling to grasp the information, or that the patient did not dare to disclose the information for fear of how the family member would react [23, 24]. We would advise that it falls on the healthcare professionals to actively invite and include family carers, apart from when the patient wishes otherwise, and also to aid and support the communication between patient and family.

It has been described that fear of taking away hope impedes healthcare professionals from initiating conversations on palliative care [10]. Sadly, several participants described precisely this – that losing hope was a consequence of discussions regarding palliative care, as this was closely associated with end of life. An aim of serious illness conversations is to re-align hope from solely focusing on survival to other desirable goals such as hope for quality time with family. Earlier introduction of serious illness conversations by trained healthcare professionals [15] and earlier integration of palliative care in parallel with active haematological care [3, 25] could be ways to achieve this. Clayton et al. [26] described three practical ways to nurture hope when talking with terminally ill cancer patients and their carers: (1) Emphasize what can be done, (2) Explore realistic goals and (3) Discuss day-to-day living. These suggestions align well with how the family carers in this study describe situations where they felt communication was positive, for example, in practical suggestions, such as how they could spend the night in the hospital with the patient. A practical area in which family carers desired more support (and typical for haematological malignancies) was in how to deal with the patient’s increased susceptibility to infection. Including the topic of infections in routine conversations and allowing the family carers to express their fears and practical struggles regarding this could be a practical and positive way of supporting them.

This study shows that family carers feel a great deal of responsibility over the care situation while they themselves are in need of support. Some found caregiving at home complicated and very stressful as a result. This is consistent with the findings of Tranberg et al., who showed that carers often feel responsible for taking on the role of project manager during the course of the illness [27]. This role included being responsible for all aspects of the patient’s care, for example, taking responsibility for medication and making appointments with the healthcare provider. The study also showed that relatives felt lonely in this role and prioritised the patient’s needs and wishes over their own. Several family carers in our study requested support from a counsellor and more focus on psycho-social issues in the conversations with healthcare professionals. This points to the need to integrate a counsellor into the care team. The counsellor then has the opportunity to complement the healthcare staff by seeing and supporting the family members and their needs.

Many carers felt that the process was unexpectedly fast and that they were unprepared for this. The fact that the nature of haematological malignancies is unpredictable (with rapid deteriorations) is known to be an impediment to transitioning to palliative care and has been repeatedly described [3, 28]. However, few studies have highlighted the effect this has on the family. In McCaughan et al. [18] interviews with bereaved carers of patients with haematological malignancies revealed several participants were unprepared for the rapid deterioration, and that they would have welcomed guidance on the possibility of rapid change, aligning with the findings in our study. This indicates a need for earlier initiation of conversations with information about severe illness and palliative care to allow relatives to prepare and plan for the future. Previous studies examining family carers and early integration of palliative care have shown that family carers exhibited less depression and a lower stress burden [29] and that family carers felt more satisfied with the care [30]. A potential improvement opportunity, therefore, is to implement routines to introduce conversations about severe illness and palliative care earlier. El-Jawahri et al. have proposed a strategy for integrating palliative care in patients with haematologic malignancies [31].

In our study, several relatives felt reassured that they had made practical preparations for the future after the patient had died. The fact that healthcare professionals can help initiate support around practical issues can help relatives prepare for the end of life. Practical information may also be an entry point for relatives to start thinking and reflecting on palliative care and the future, as suggested by Clayton et al. [26] and as supported by our findings, where several relatives felt that such information was easier to absorb.

### Strengths and weaknesses

This is a small study, with only 9 participants, due to ethical and pragmatic restraints. We did not aim to reach data saturation in such a complex topic as this. Nevertheless the emerging themes described here were clear and consistent through the interviews, though further interviews undoubtedly would increase the complexity of the picture and probably introduce further themes worthy of exploring. The participants were all Swedish and their relatives had all been treated at the same hematological clinic, which limits the perspectives captured in this study. Further studies in other cultural and socio-economic settings are required to establish the transferability of these findings.

As the present study is based on voluntary participation, there is a risk of selection bias. Those who chose to participate may have been particularly motivated to share their experiences, which may have affected the results. In cases where the patient requested confidentiality, family carers could not be included, which means that this perspective was not captured. Information about the study was sent to the patient’s estate, which may have meant not all potential relatives received information about the opportunity to participate in the study. Participants were allowed to choose the location of the interview, which may have contributed to a safer interview situation and increased the likelihood of their sharing their experiences as truthfully as possible.

## Conclusion

This study shows that haematological care could gain a lot by focusing more on communication with family carers. Many family carers take a great deal of responsibility for the care of the patient and need to be given support to be able to do this in the best way. To improve the situation for family carers, they need to be invited to discussions and receive regular updates on the current condition. The information should be truthful and provided in a way that allows the family carers to maintain hope in a realistic manner, without inducing false hopes. Providing written information and the opportunity for a follow-up conversation soon after difficult news, as well as routinely raising the topic of how to handle the risk of infections, have been given as practical suggestions. Procedures should be put in place to initiate conversations about serious illness and palliative care earlier so that families have the opportunity to prepare for the future.

## Supplementary Information

Supplementary Material 1.

## Data Availability

Original interview data can not be publicly shared due to confidentiality principles under Swedish law. Original data can be provided upon reasonable request with ethical approval.
