# Supplementary material for: Family carers’ experiences of serious illness conversations in haematological malignancies – a qualitative interview study
Source: BMC Palliat Care. 2026 Jul 4;25:197. doi: 10.1186/s12904-026-02214-w (PMC13343705; doi:10.1186/s12904-026-02214-w)
Supplement: Supplementary file 1 — Supplementary Material 1. [file 12904_2026_2214_MOESM1_ESM.docx]

**Demographic information collected at start of interview**

Relationship with the patient

Patient and respondent gender and age

Patient and respondent educational background

Patient and respondent family situation

Patient and respondent employment situation

Patient and respondent country of birth

Patient illness

Time in last type of care for patient

**Interview guide for family carer interviews**

**Introduction:**

We will talk about conversations you had about your family member’s illness, about their care and about how you and your family member thought about the future.

Would you like to tell us about conversations you had with health professionals about your family member’s illness?

*Additional questions as needed:*

- What has your family member been told in the past about the future? (How do you feel about those messages today?)

- In what way have the conversations touched on forecasting? (How do you think about it now?)

- Were there opportunities for being cured?

- Do you know why your family member received their treatment, how it would help?

- Were you informed about the advantages and disadvantages of the treatment, any

side effects? (What does it mean? Can you tell me more?)

- What does palliative care mean to you?

- How would you prefer these conversations to be handled by health professionals?

- When would it be best to introduce such talks?

- How would they ideally be introduced?

- What do you see as most desirable in terms of conversations about serious illness and palliative care?

- Do you have any advice or suggestions for staff on how to structure these conversations?

- What did you want to know and understand?

- What do you wish they would have talked about?

- In the situation you are in now, what would you wish for?

- Who would you have liked to talk to about it?

- Is there anything that has made you feel unsafe/secure? (What does it mean? Can you tell me more?)

- Does the location matter for the conversation with the healthcare professional?
